# Metabolomic profiling of cancer-related fatigue involved in cachexia and chemotherapy

**DOI:** 10.1038/s41598-024-57747-y

**Published:** 2024-04-09

**Authors:** Yuki Okinaka, Susumu Kageyama, Toshiyuki Goto, Masahiro Sugimoto, Atsumi Tomita, Yumi Aizawa, Kenichi Kobayashi, Akinori Wada, Akihiro Kawauchi, Yosky Kataoka

**Affiliations:** 1 Department of Urology, Shiga University of Medical Science, Shiga, 520-2192 Japan; 2 RIKEN Center for Biosystems Dynamics Research, Hyogo, 650-0047 Japan; 3 Institute of Medical Science, Tokyo Medical University, Tokyo, 160-8402 Japan; 4 Institute for Advanced Biosciences, Keio University, Yamagata, 997-0052 Japan; 5 Graduate School of Science, Technology and Innovation, Kobe University, Hyogo, 650-0047 Japan

**Keywords:** Biomarkers, Diagnostic markers, Metabolomics, Oncology

## Abstract

Patients with advanced cancer are frequently burdened with a severe sensation of fatigue called cancer-related fatigue (CRF). CRF is induced at various stages and treatments, such as cachexia and chemotherapy, and reduces the overall survival of patients. Objective and quantitative assessment of CRF could contribute to the diagnosis and prediction of treatment efficacy. However, such studies have not been intensively performed, particularly regarding metabolic profiles. Here, we conducted plasma metabolomics of 15 patients with urological cancer. The patients with and without fatigue, including those with cachexia or chemotherapy-induced fatigue, were compared. Significantly lower concentrations of valine and tryptophan were observed in fatigued patients than in non-fatigued patients. In addition, significantly higher concentrations of polyamine pathway metabolites were observed in patients with fatigue and cachexia than in those without cachexia. Patients with exacerbated fatigue due to chemotherapy showed significantly decreased cysteine and methionine metabolism before chemotherapy compared with those without fatigue exacerbation. These findings suggest that plasma metabolic profiles could help improve the diagnosis and monitoring of CRF.

## Introduction

Patients with advanced cancer are frequently burdened with a severe sensation of fatigue called cancer-related fatigue (CRF). Approximately 75% of patients with metastatic cancer and 70–100% with cachexia experience CRF^1–3^. Cancer treatments, including radiation and chemotherapy, were also found to induce CRF in 80–84% of patients^1^. CRF significantly interferes with physiological function through physical, emotional, and cognitive exhaustion in patients^4^ and reduces their quality of life^5,6^. For example, among patients with cancer complaining of fatigue during chemotherapy, 91% showed difficulty with leading normal lives and 88% were forced to alter their daily routines^7^. Furthermore, CRF often interferes with treatment completion, which reduces the overall survival of patients^7,8^. Thus, CRF is a critical problem for the treatment and well-being of patients with cancer; however, it is not well understood.

Questionnaire-based CRF assessments, such as the Numerical Rating Scale^9^, Brief Fatigue Inventory^10^, Cancer Fatigue Scale^11^, and 13-item Functional Assessment of Chronic Illness Therapy-Fatigue (FACIT-F)^12^, have been used to evaluate CRF in patients. However, a gap between such subjective evaluations by patients and physicians is often observed^13^, which prevents treatment optimization. Owing to such inadequate and controversial CRF assessments^14^, the development of more objective methods for evaluating CRF is required.

Elevated serum levels of inflammatory and anti-inflammatory cytokines, including interleukin 6, tumor necrosis factor-α, and interleukin 1 receptor antagonist, have been reported in patients with CRF^15–17^. Serum C-reactive protein (CRP) levels have also been reported to be associated with CRF in patients or survivors of testicular, breast, or other advanced cancers^18–20^. However, inconsistent results have been reported for cytokines^18,21,22^ and CRP^17,23^.

Non-inflammatory molecules have also been identified as potential CRF biomarkers. Lower serum hemoglobin and albumin levels have been reported in patients with CRF^24,25^. Another report demonstrated a moderate association between CRF and hemoglobin levels, but not with albumin^26^. However, hemoglobin level was no longer a significant predictor when the effect of inflammation was removed^27^. These reports indicate that hemoglobin is not a reliable CRF biomarker. Thus, effective biomarkers for CRF are yet to be identified.

Studies on the pathophysiology of fatigue have revealed characteristic alteration in metabolism^28,29^, variations in microbial species^30^, and dysfunction of the neuro-immuno-endocrine system^31,32^. Metabolome analysis was conducted in patients with myalgic encephalomyelitis/chronic fatigue syndrome (CFS). Fatigue-induced animal models showed similar plasma metabolic profiles. Significant changes in intermediate metabolite concentrations in the tricarboxylic acid (TCA) and urea cycles were observed^29^. A fatigued rat model showed decreased energy metabolism in response to changes in the urea cycle and amino acids, including branched-chain amino acids (BCAAs)^28^. These findings suggest that blood metabolites are effective biomarkers of fatigue. Recently, metabolomic analyses have been used to identify CRF biomarkers. Metabolic pathways involved in glutathione, glutamine, and glutamate metabolism are associated with CRF in patients with colorectal cancer^33^ and those involved in sphingolipid metabolism, histidine metabolism, and cysteine and methionine metabolism in patients with various carcinomas^34^.

Patients with cancer often suffer from CRF under cachexia or various treatments, including chemotherapy^3^. A previous study using animal models of cancer- and chemotherapy-induced cachexia reported similar alterations in plasma metabolic profiles during the TCA cycle and β-oxidation^35^. However, no metabolic profiling studies have been conducted on patients with CRF considering such conditions including cachexia or chemotherapy. In this study, we performed both questionnaire-based CRF assessment and plasma metabolome analysis in patients with urological cancers, including urothelial and prostate cancer. This study analyzed the metabolic profiles of patients with CRF and cachexia. The relationship between chemotherapy-related fatigue and metabolic profiles was also analyzed.

## Results

We analyzed FACIT-F fatigue scores and plasma metabolomic profiles of 15 patients with urological cancer. Patient demographics are shown in Table 1. Of the five patients in the cachexia group, three had urothelial cancer and two had prostate cancer, and the other 10 patients in the non-cachexia group had urothelial cancer. All patients in the non-cachexia group (10 patients) received chemotherapy; nine patients received a regimen including a platinum antitumor agent (gemcitabine plus cisplatin or gemcitabine plus carboplatin) and one patient received gemcitabine plus paclitaxel. Of the 15 patients, 10 were classified into the fatigued group and five into the non-fatigued group based on the questionnaire-based CRF assessment. All patients with cachexia were included in the fatigued group. The primary endpoint of this study was to assess the metabolic profiles of patients with CRF. In addition to the primary endpoint, the secondary endpoint involves revealing the metabolome profile before chemotherapy in both the groups experiencing exacerbated fatigue and not.

**Table 1:** Participants’ characteristics.

Characteristic	Non-cachexia		Cachexia
	Non-fatigued (n = 5)	Fatigued (n = 5)	Fatigued (n = 5)
Age (years)			
Median	62	70	67
Range	57–76	66–82	50–70
Sex (n) (male/female)	5/0	4/1	5/0
Diagnosis (n)			
Urothelial cancer	5	5	3
Prostate cancer	0	0	2
Metastasis (yes/no)	4/1	5/0	5/0
Chemotherapy			
GEM/CDDP	4	2	
GEM/CBDCA	1	2	
GEM/PTX	0	1	

*GEM* Gemcitabine, *CDDP* Cisplatin, *CBDCA* Carboplatin, *PTX* Paclitaxel.

### Metabolites involved in cancer-related fatigue (comparison 1)

**Figure 1:**
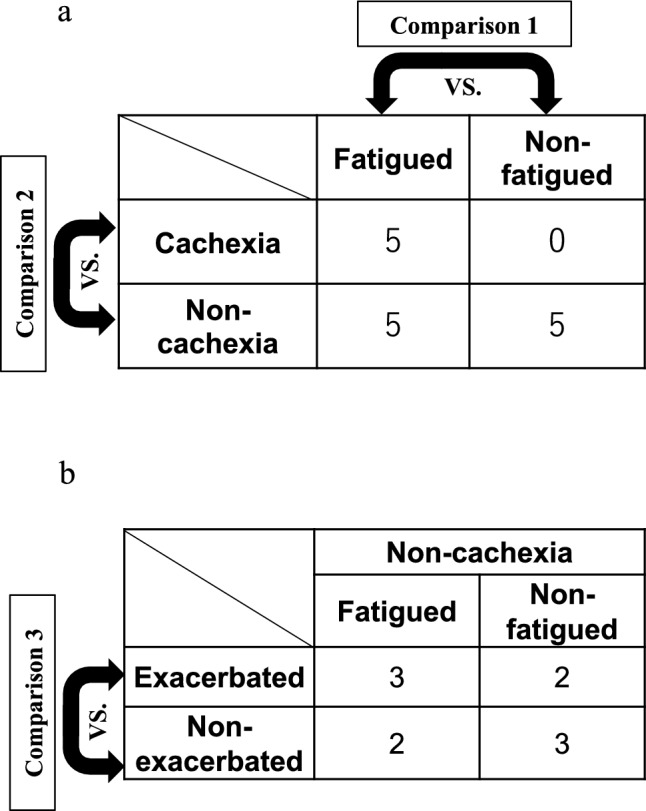
Study design. (**a**) Patients were divided into non-fatigued (n = 5) and fatigued (n = 10) groups using the 13-item Functional Assessment of Chronic Illness Therapy-Fatigue (FACIT-F) scores. Further, patients in the fatigued group were divided into non-cachexia (n = 5) and cachexia (n = 5) groups using the definition of the cachexic condition. Two types of comparison in plasma metabolomic profiles were conducted: comparison 1, non-fatigued group vs. fatigued group; comparison 2, non-cachexia group vs. cachexia group. (**b**) All patients without cachexia underwent chemotherapy. Those patients were divided into exacerbated (n = 5) and non-exacerbated (n = 5) groups using the change in FACIT-F fatigue scores observed before and after chemotherapy. Comparison in plasma metabolomic profiles was conducted between the exacerbated fatigue group vs. the non-exacerbated fatigue group (comparison 3).

**Figure 2:**
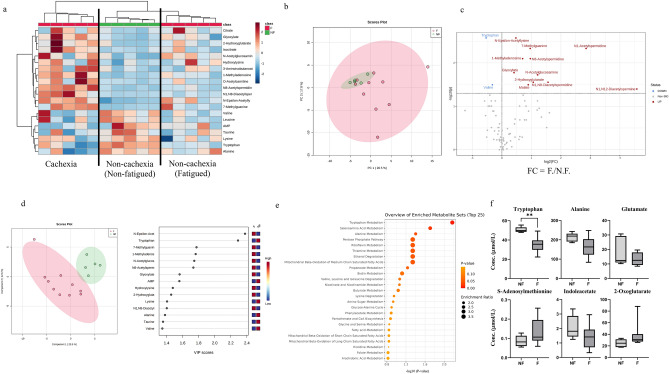
Plasma metabolites in the fatigued and non-fatigued groups. (**a**) Hierarchical clustering heatmap analysis of plasma metabolomic data. Metabolite concentrations were normalized by dividing each concentration value with the average concentration measured across all patients. Higher concentrations compared with that of the average were represented in red, lower concentrations in blue, and concentrations similar to that of the average represented in white. (**b**) Score plot of principal component analysis (PCA) of plasma metabolite. The contribution ratio of PC1 and PC2 were 26.5% and 17.8%, respectively. Red represents the fatigue group and green the non-fatigue group. (**c**) Volcano plots showing differences in metabolite concentrations between the fatigued and non-fatigued groups. The X- and Y-axes indicate the log_2_ fold change (fatigued/non-fatigued) and − log_10_ *P*-values (Mann–Whitney *U* test), respectively. (**d**) Score plots of partial least squares-discriminant analysis (PLS-DA) (left-hand figure). The X- and Y-axes indicate the first and second components. Quantile normalization was performed on each sample, followed by autoscaling of the metabolite concentrations to eliminate sample-dependent bias. Red represents the fatigue group and green the non-fatigue group. Variable importance in projection (VIP) scores showing the top 15 metabolites (right-hand figure). Higher concentrations compared with that of the average were represented in red and lower concentrations in blue. (**e**) Metabolic pathway-based analysis showing the top 25 enriched metabolite sets. The color intensity represents *P*-values, whereas the size of the circles represents the enrichment ratio. (**f**) Box plots of each metabolite concentration in the tryptophan metabolic pathway. Horizontal lines of the box indicate 0, 25, 50, 75, and 100% of the data. The Y-axis indicates metabolite concentrations (μM). *P < 0.05, **P < 0.01 (Mann–Whitney *U* test).

In this study, liquid chromatography time-of-flight mass spectrometry (LC-TOF-MS) was used to successfully identify and quantify 151 metabolites in plasma samples collected from the patients with CRF. We compared the plasma metabolite concentrations between the fatigued group, including the cachexic (five patients) and non-cachexic patients (five patients), and the non-fatigued group (five patients) to clarify the influence of CRF on the metabolites (Fig. 1). A hierarchical clustering heatmap analysis provided an overview of the concentration patterns among these three groups (Fig. 2a). Each sample was clearly clustered into each group. Principal component analysis (PCA) showed a larger variety in the metabolomic profile of the fatigued group than that of the non-fatigued group (Fig. 2b).

Volcano plots revealed 13 plasma metabolites with significant differences between the fatigued and non-fatigued groups (Fig. 2c). Of these, 11 showed higher concentrations and two showed lower concentrations in the fatigued group than in the non-fatigued group (*P* < 0.05, Mann–Whitney *U* test). The metabolites with higher concentrations in the fatigued group were *N*^1^,*N*^12^-diacetylspermine, *N*^1^-acetylspermidine, *N*^1^,*N*^8^-diacetylspermidine, *N*^8^-acetylspermidine, 2-hydroxyglutarate, 7-methylguanine, 1-methyladenosine, *N*-acetylglucosamine, glyoxylate, *N*^6^-acetyllysine, and malate. The metabolites with lower concentrations in the fatigued group were valine and tryptophan. In comparing the two groups, Cohen’s d values exceeded 0.8 for all comparisons except *N*^1^,*N*^12^-diacetylspermine (Cohen’s d = 0.56) and *N*^1^-acetylspermidine (Cohen’s d = 0.62). Note that *N*^6^-acetyllysine and tryptophan showed significantly different concentrations in the fatigued/non-fatigued groups, with *Q*-value, i.e., false discovery rate (FDR)-corrected *P*-value (Table 2 and Supplementary Fig. S1). The leucine concentration was also lower in the fatigued group than in the non-fatigued group (*P* = 0.055) (Table 2).

**Table 2:** Blood metabolite levels between the non-fatigued and fatigued groups.

Metabolites	Non-fatigued		Fatigued		F.C.^a^	P-value^b^	Q-value^c^
	Average	S.D.	Average	S.D.			
Tryptophan	50.6	2.92	34.9	7.72	0.69	0.0027	0.10
*N*-Epsilon-Acetyllysine	0.393	0.0267	0.546	0.0681	1.4	0.0027	0.10
7-Methylguanine	0.133	0.0130	0.259	0.103	1.9	0.0047	0.10
*N*^1^-Acetylspermidine	0.0489	0.0138	0.354	0.605	7.2	0.0047	0.10
1-Methyladenosine	0.144	0.0135	0.234	0.0742	1.6	0.0080	0.11
*N*^8^-Acetylspermidine	0.047	0.00716	0.0922	0.0380	2.0	0.0080	0.11
Glyoxylate	44.7	4.06	59.4	14.3	1.3	0.017	0.20
*N*-Acetylglucosamine	0.0282	0.0171	0.0653	0.0291	2.3	0.019	0.20
2-Hydroxyglutarate	3.79	1.87	7.91	4.31	2.1	0.028	0.23
*N*^1^, *N*^8^-Diacetylspermidine	0.00843	0.00160	0.0249	0.0188	3.0	0.028	0.23
Valine	221	29.7	177	49.5	0.80	0.032	0.23
Malate	14.7	4.04	27.3	18.6	1.9	0.032	0.23
*N*^1^, *N*^12^-Diacetylspermine	0.00527	0.00100	0.126	0.267	23.8	0.040	0.26
Leucine	142	26.3	107	42.5	0.75	0.055	0.32
Isocitrate	12.5	2.16	20.5	9.6	1.6	0.055	0.32
Taurine	26.1	5.74	20.4	5.69	0.8	0.075	0.32
Lysine	95.2	6.90	81.5	14.9	0.86	0.075	0.32
Citrate	56.5	16.0	96.8	50.0	1.7	0.075	0.32
Pipecolate	2.14	0.639	4.94	4.85	2.3	0.075	0.32
AMP	3.04	1.09	2.02	0.71	0.66	0.075	0.32

S.D. and F.C. indicate standard deviation and fold change, respectively.

^a^Fatigued/non-fatigued.

^b^P-values for each metabolite were calculated using the Mann–Whitney *U* test.

^c^Q-value indicates the P-value corrected by false discovery rate.

Partial least squares-discriminant analysis (PLS-DA) was conducted to evaluate the discrimination ability of the overall metabolomic data between the two groups (Fig. 2d). The fatigued group was separated from the non-fatigued group in this analysis. Metabolites that contributed to discrimination were ranked with high variable importance in projection (VIP) scores (Fig. 2d). Metabolic pathway-based analysis showed that tryptophan metabolism contributed the most to the differences observed between the two groups (Fig. 2e). The concentrations of six metabolites in the tryptophan metabolic pathway were observed in the fatigued/non-fatigued groups (Fig. 2f). These findings indicate that patients with CRF have different metabolomic profiles than in those without CRF.

In the fatigued group, five patients with cachexia were included. Thus, we compared metabolites between the fatigued group without cachexia and the non-fatigued group due to the exclusion of the impact of cachexia on plasma metabolites (Supplementary Fig. S2). Concentrations of *N*-acetylglucosamine and *N*^6^-acetyllysine were higher in the fatigued group without cachexia than in the non-fatigued group. Valine, leucine, and tryptophan concentrations remained lower even in the fatigued group without cachexia, as shown in the fatigued group containing patients with cachexia (Supplementary Fig. S2a). Calculating Cohen’s d values for all of these metabolites showed values exceeding 0.8. PLS-DA was used to evaluate the discrimination ability of the overall metabolomic data between the two groups (Supplementary Fig. S2b). The fatigued group without cachexia was also separated from the non-fatigued group by using the calculated VIP scores (Supplementary Fig. S2b). As with the previous result including the cachexia group, the metabolic pathway-based analysis showed that tryptophan metabolism contributed most potently to the difference observed between the two groups (Supplementary Fig. S2c). The concentrations of six metabolites in the tryptophan metabolic pathway in the fatigue without cachexia/non-fatigued groups are shown in Supplementary Fig. S2d. These results indicate that valine, leucine, tryptophan,* N*-acetylglucosamine, and *N*^*6*^-acetyllysine metabolites still showed different concentrations between the patients with CRF and those without CRF, even if the impact of cachexia was excluded.

### Metabolites involved in cachexia (comparison 2)

**Figure 3:**
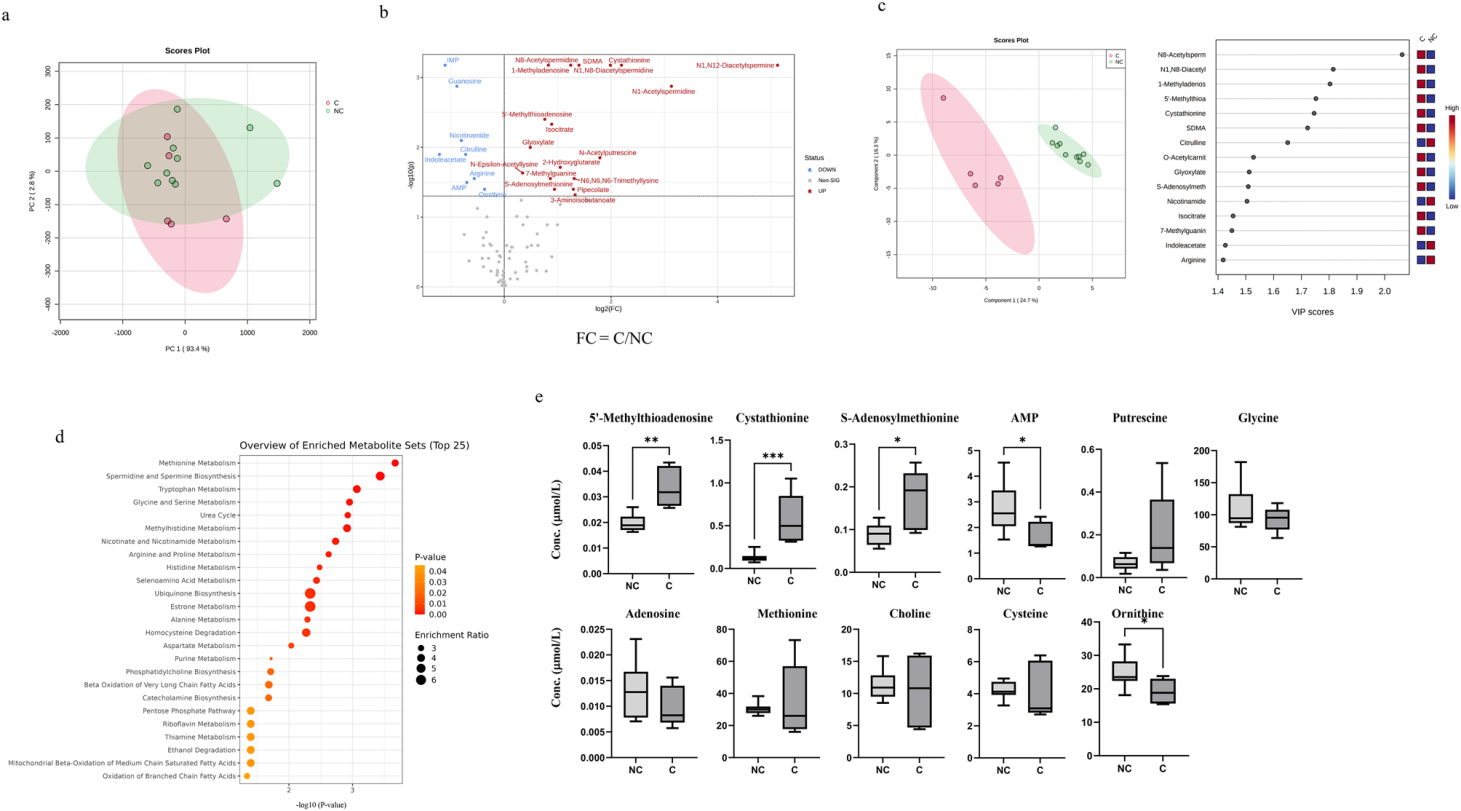
Plasma metabolites in the cachexia and non-cachexia groups. (**a**) Score plot of the principal component analysis (PCA) of plasma metabolites. Contribution ratio of PC1 and PC2 were 93.4% and 2.8%, respectively. Red represents the cachexia group and green the non-cachexia group. (**b**) Volcano plots showing differences in individual metabolite concentrations between the cachexia and non-cachexia groups. The X- and Y-axes indicate the log_2_ fold change (cachexia/non-cachexia) and − log_10_ *P*-values (Mann–Whitney *U* test), respectively. (**c**) Score plots of partial least squares-discriminant analysis (PLS-DA) (left-hand figure). The X- and Y-axes indicate the first and second components. Quantile normalization was performed on each sample, followed by autoscaling of the metabolite concentrations to eliminate sample-dependent bias. Red represents the cachexia group and green the non-cachexia group. Variable importance in projection (VIP) scores showing the top 15 metabolites (right-hand figure). Higher concentrations compared with that of the average were represented in red and lower concentrations in blue. (**d**) Metabolic pathway-based analysis showing the top 25 enriched metabolite sets. The color intensity represents *P*-values, whereas the size of the circles represents the enrichment ratio. (**e**) Box plots of each metabolite concentration in methionine metabolism and spermidine and spermine biosynthesis. Horizontal lines of the box indicate 0, 25, 50, 75, and 100% of the data. The Y-axis indicates metabolite concentrations (μM). C., cachexia group; N.C., non-cachexia group. *P < 0.05, **P < 0.01 (Mann–Whitney *U* test).

We compared plasma metabolite concentrations between the cachexia (five patients) and non-cachexia groups, including fatigued (five patients) and non-fatigued patients (five patients), to clarify the influence of cachexia on these metabolites (Fig. 1). PCA demonstrated a deviation in the metabolomic profile of the non-cachexia group from that of the cachexia group (Fig. 3a). Metabolites showing significantly different plasma concentrations between the cachexia and non-cachexia groups are shown in volcano plots (Fig. 3b). Metabolites showing higher concentrations in the cachexia group compared with those in the non-cachexia group were as follows: *N*^1^,*N*^12^-diacetylspermine, *N*^1^-acetylspermidine, *N*^1^,*N*^8^-diacetylspermidine, *N*^8^-acetylspermidine, *N*-acetylputrescine, cystathionine, *S*-adenosylmethionine, 5′-methylthioadenosine, *N*^6^,*N*^6^,*N*^6^-trimethyllysine, *N*^6^-acetyllysine, pipecolate, 2-hydroxyglutarate, isocitrate, and symmetric dimethylarginine (SDMA). Additionally, the cachexia group had higher concentrations of 1-methyladenosine, 7-methylguanine, 3-aminoisobutanoate, and glyoxylate. Meanwhile, the cachexia group showed lower concentrations of adenosine 5′-monophosphate, inosine 5′-monophosphate (IMP), guanosine, citrulline, arginine, ornithine, nicotinamide, and indole acetate. In all of these metabolites, the Cohen’s d values exceeded 0.8. Note that *N*^1^,*N*^12^-diacetylspermine, *N*^1^-acetylspermidine, *N*^1^,*N*^8^-diacetylspermidine, *N*^8^-acetylspermidine, cystathionine, 5′-methylthioadenosine, isocitrate, SDMA, 1-methyladenosine, IMP, and guanosine showed significantly different concentrations in the cachexia/non-cachexia groups with Q-value (Supplementary Fig. S3).

PLS-DA was used to evaluate the discriminatory ability of the overall metabolomic data between the two groups (Fig. 3c). In this analysis, the cachexia group was separated from the non-cachexia group. Additionally, we identified metabolites with high VIP scores (Fig. 3c). The metabolic pathway-based analysis showed that methionine metabolism and spermidine and spermine metabolism contributed significantly to the differences observed between the two groups (Fig. 3d). Concentrations of 11 metabolites in methionine metabolism and spermidine and spermine metabolism were observed in the cachexia/non-cachexia groups (Fig. 3e). The cachexia group exhibited a unique metabolomic profile.

**Figure 4:**
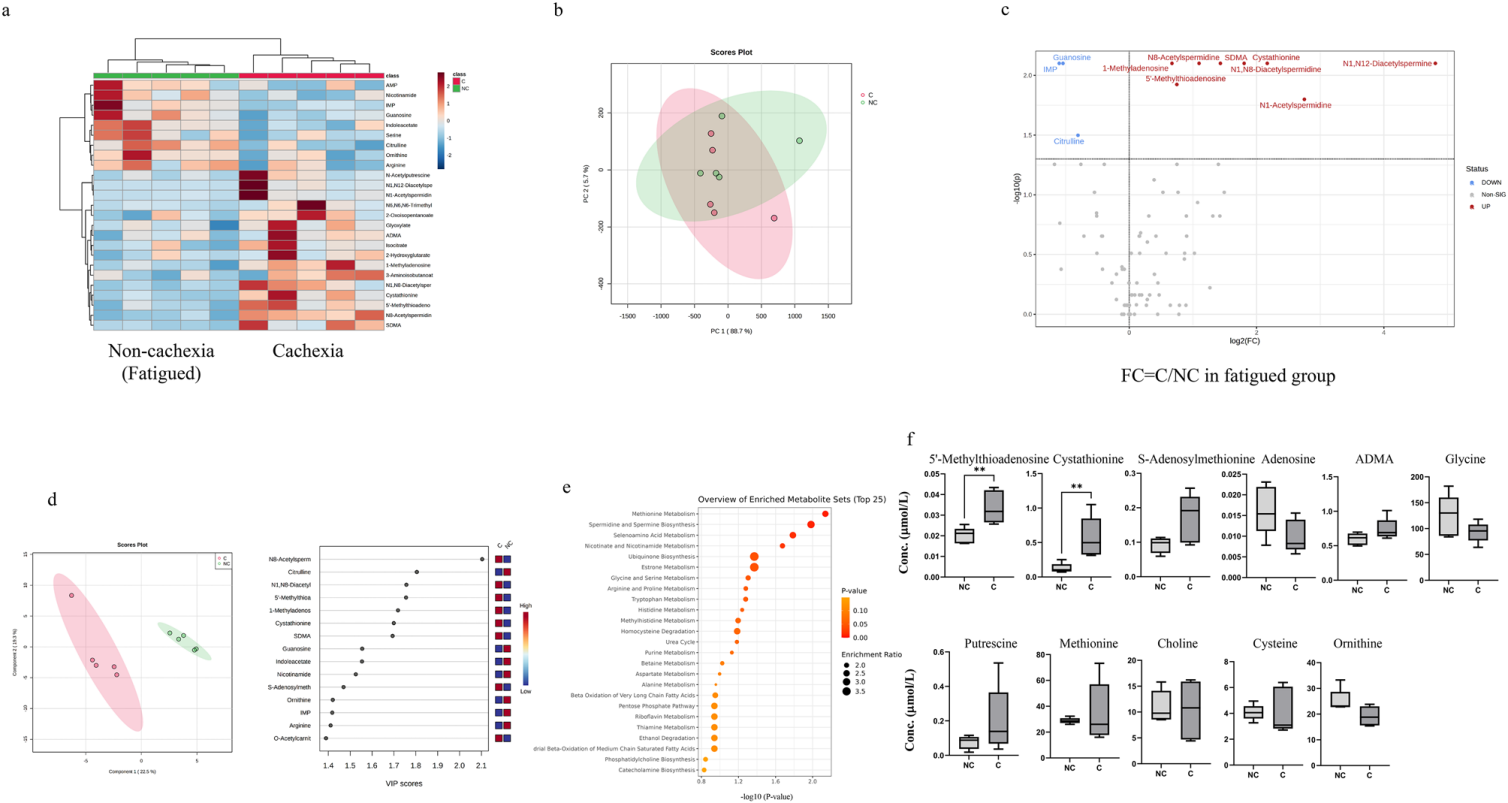
Plasma metabolites in the cachexia and non-cachexia groups with fatigue groups. (**a**) Hierarchical clustering heatmap analysis of plasma metabolomic data. Metabolite concentrations were normalized by dividing each concentration value with the average concentration measured across all patients. Higher concentrations compared with that of the average were represented in red, lower concentrations in blue, and concentrations similar to that of the average represented in white. (**b**) Score plot of the principal component analysis (PCA) of plasma metabolites. Contribution ratio of PC1 and PC2 were 88.7% and 5.7%, respectively. Red represents the cachexia group and green the non-cachexia with fatigue group. (**c**) Volcano plots showing differences in metabolite concentrations between the cachexia and non-cachexia with fatigue groups. The X- and Y-axes indicate the log_2_ fold change (cachexia/non-cachexia with fatigue) and –log_10_ *P*-values (Mann–Whitney *U* test), respectively. (**d**) Score plots of partial least squares-discriminant analysis (PLS-DA) (figure on the left). The X- and Y-axes indicate the first and second components. Quantile normalization was performed on each sample, followed by autoscaling of the metabolite concentrations to eliminate sample-dependent bias. Red represents the cachexia group and green the non-cachexia with fatigue group. Variable importance in projection (VIP) scores showing the top 15 metabolites (right-hand figure). Higher concentrations compared with that of the average were represented in red and lower concentrations in blue. (**e**) Metabolic pathway-based analysis showing the top 25 enriched metabolite sets. The color intensity represents *P*-values, whereas the size of the circles represents the enrichment ratio. (**f**) Box plots of each metabolite concentration in methionine metabolism and spermidine and spermine biosynthesis. Horizontal lines of the box indicate 0, 25, 50, 75, and 100% of the data. The Y-axis indicates metabolite concentrations (μM). C., cachexia group; N.C., non-cachexia with fatigue group. *P < 0.05, **P < 0.01 (Mann–Whitney *U* test).

Although all patients underwent CRF in the cachexia group, only five showed CRF in the non-cachexia group. Thus, we compared metabolites between the cachexia and non-cachexia groups with CRF due to the exclusion of the impact of CRF on plasma metabolites (Fig. 4). A hierarchical clustering heatmap analysis revealed the relative abundance of metabolites between the two groups (Fig. 4a). PCA demonstrated a deviation in the metabolomic profile of the cachexia group compared with that of the non-cachexia group with CRF (Fig. 4b). The cachexia group showed higher concentrations compared with those of the non-cachexia group with CRF in eight metabolites, including *N*^8^-acetylspermidine, *N*^1^,*N*^8^-diacetylspermidine, *N*^1^-acetylspermidine, *N*^1^,*N*^12^-diacetylspermine, cystathionine, 5′-methylthioadenosine, SDMA, and 1-methyladenosine. Meanwhile, the cachexia group showed lower concentrations in three metabolites, including IMP, guanosine, and citrulline (Fig. 4c). In all of these metabolites, the Cohen’s d values exceeded 0.8. The concentrations of these metabolites showed no significant differences from the FDR-corrected *P*-value of the PLS-DA, which evaluated the discrimination ability of the overall metabolomic data between the two groups (Fig. 4d). The cachexia group was separated from the non-cachexia group with CRF in this analysis. Moreover, metabolites with high VIP scores were identified (Fig. 4d). Metabolic pathway-based analysis showed that methionine metabolism and spermidine and spermine metabolism contributed significantly to the differences observed between the two groups, similar to that of the analysis of the cachexia and non-cachexia groups containing non-fatigued patients (Fig. 4e). The concentrations of 11 metabolites in methionine metabolism and spermidine and spermine metabolism are shown in the cachexia/non-cachexia with CRF groups in Fig. 4f. Among the patients with CRF, the cachexia group showed different profiles from those of the non-cachexia group.

### Metabolites involved in chemotherapy-induced cancer-related fatigue (comparison 3)

**Figure 5:**
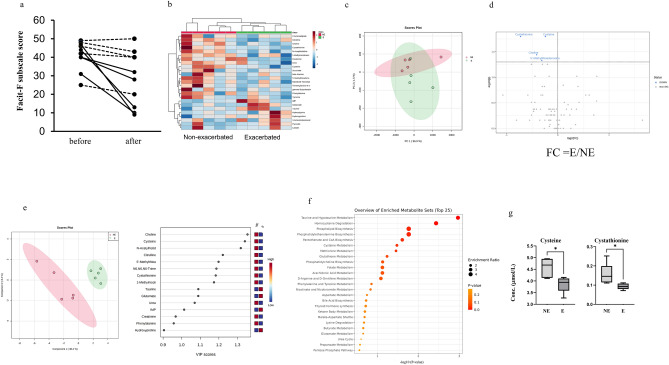
Plasma metabolites in the exacerbated and non-exacerbated groups in chemotherapy. (**a**) The 13-item Functional Assessment of Chronic Illness Therapy-Fatigue (FACIT-F) subscale scores. Grey broken lines indicate data in the non-exacerbated group (n = 5) and black bold lines indicate those in the exacerbated group (n = 5). (**b**) Hierarchical clustering heatmap analysis of the plasma metabolomic data. Metabolite concentrations were normalized by dividing each concentration value with the average concentration measured across all patients. Higher concentrations compared with those of the average were represented in red, lower concentrations in blue, and concentrations similar to that of the average represented in white. (**c**) Score plot of the principal component analysis (PCA) of plasma metabolites. Contribution ratio of PC1 and PC2 were 96.9% and 1.5%, respectively. Red represents the exacerbated group and green the non-exacerbated group. (**d**) Volcano plots showing differences in metabolite concentrations between the exacerbated and non-exacerbated groups. The X- and Y-axes indicate the log_2_ fold change (exacerbated/non-exacerbated) and − log_10_ *P*-values (Mann–Whitney *U* test), respectively. (**e**) Score plots of partial least squares-discriminant analysis (PLS-DA) (left-hand figure). The X- and Y-axes indicate the first and second components. Quantile normalization was performed on each sample, followed by autoscaling of the metabolite concentrations to eliminate sample-dependent bias. Red represents the exacerbated group and green the non-exacerbated group. Variable importance in projection (VIP) scores showing the top 15 metabolites (right-hand figure). Higher concentrations compared with that of the average were represented in red and lower concentrations in blue. (**f**) Metabolic pathway-based analysis showing the top 25 enriched metabolite sets. The color intensity represents *P*-values, whereas the size of the circles represents the enrichment ratio. (**g**) Box plots of each metabolite concentration in homocysteine degradation. Horizontal lines of the box indicate 0, 25, 50, 75, and 100% of the data. The Y-axis indicates metabolite concentrations (μM). E., exacerbated group; N.E., non-exacerbated group.

In this study, 10 patients underwent chemotherapy, and the impact of chemotherapy on fatigue sensation was analyzed. FACIT-F fatigue scores before and after a series of chemotherapy sessions showed that five patients suffered from exacerbated fatigue after chemotherapy (exacerbated group) whereas the other five patients did not (non-exacerbated group) (Fig. 1). These chemotherapy-induced changes in the FACIT-F fatigue scores were not associated with the scores before chemotherapy (Fig. 5a). We compared the plasma metabolites before and after chemotherapy in both the exacerbated and non-exacerbated groups to identify metabolites that may predict the occurrence of chemotherapy-induced CRF. We identified differences in the concentrations of metabolites before chemotherapy between the two groups, and the results are presented below. Hierarchical clustering heatmap analysis was used to visualize the metabolite patterns between the exacerbated and non-exacerbated groups (Fig. 5b). PCA was conducted to determine the distribution of metabolomic profiles between the two groups (Fig. 5c). The PCA revealed a higher variety in metabolic profiles in the non-exacerbated group than in the exacerbated group (Fig. 5c). There were four metabolites, such as cysteine, cystathionine, choline, and 5′-methylthioadenosine, showing significantly different plasma concentrations between the exacerbated and non-exacerbated groups, as shown in the volcano plots (Fig. 5d). The Cohen’s d values for all four metabolites were above 0.8. The concentrations of these metabolites showed no significant differences with the *P*-value corrected via FDR. Furthermore, the concentrations of the four metabolites were not altered by chemotherapy in either group.

PLS-DA was used to evaluate the discrimination ability of the overall metabolomic data between the two groups (Fig. 5e). The exacerbated group was separated from the non-exacerbated group in this analysis. In addition, we identified metabolites with high VIP scores (Fig. 5e). Metabolic pathway-based analysis showed that taurine and hypotaurine metabolism and homocysteine degradation contributed significantly to the differences observed between the two groups (Fig. 5f). The concentrations of the two metabolites in homocysteine degradation are shown in the exacerbated/non-exacerbated groups in Fig. 5g. The exacerbated group showed a significant decrease in the levels of four metabolites because of chemotherapy, including lactate, citrate, citrulline, and trimethylamine N-oxide. This group exhibited a significant increase in urea levels after chemotherapy. There were no significant changes in the metabolite levels after chemotherapy in the non-exacerbated group; however, the exacerbated group exhibited specific metabolic changes. These findings indicate the possibility of predicting fatigue exacerbation due to chemotherapy before its implementation.

## Discussion

CRF has a negative impact on many patients with advanced cancer. However, objective biomarkers of CRF, including those by cachexia and chemotherapy, have not been developed. In the present study, we performed metabolomic analyses of the plasma obtained from patients with and without CRF, including those with cachexia or chemotherapy-induced CRF. To the best of our knowledge, this is the first study to analyze the metabolic profiles of CRF by such causes.

Valine, a BCAA, and tryptophan, an intermediate metabolite of the nicotinamide adenine dinucleotide (NAD) synthesis pathway^36^, in the plasma showed lower concentrations in the fatigued group than in the non-fatigued group (Fig. 2). Such a decrease in BCAAs has also been reported in a previous study on cancer-induced cachexia in experimental animals^35^. Notably, even after excluding the impact of cachexia, the fatigued group showed decreased levels of BCAAs, such as valine, leucine, and tryptophan (Table 2). A previous study reported a decrease in BCAAs in muscles and mental fatigue^37,38^. Tryptophan is an important metabolite in the NAD synthesis pathway. NAD+ is an essential cofactor for mitochondrial energy production^39^. It was reported that the levels of adenosine 5′-triphosphate in the liver and skeletal muscles were significantly decreased in fatigued models in rats^28^. Such decreases in plasma BCAAs and tryptophan levels in the fatigued group of patients in the present study might indicate a functional deterioration in muscle synthesis and mitochondrial energy production. Although diet and eating habits influenced the serum metabolome containing amino acids^40,41^, we did not analyze usual eating habits. Future research demands a detailed examination of diet and eating habits.

Exercise-induced fatigue in the central nervous system has been reported to be related to the concentration of serotonin in the brain^42^. Serotonin concentration in the brain is affected by the ratio of tryptophan to BCAAs in the blood; a decrease in BCAAs increases the transfer of tryptophan to the brain, resulting in the increase in serotonin concentration in the brain^43,44^. Oral administration of BCAAs in rats decreased both tryptophan and serotonin levels in the brain 1 h after administration^45^. In addition, in human subjects, ingestion of BCAAs increases plasma BCAA concentrations and improves mental fatigue^46^. In the present study, BCAA concentrations were lower in patients with fatigue, indicating the possibility that BCAA intake may improve CRF.

In this study, the concentration of metabolites of the polyamine metabolic pathway, including *N*^1^,*N*^12^-diacetylspermine, *N*^1^-acetylspermidine, *N*^1^,*N*^8^-diacetylspermidine, acetylspermidine, and *N*-acetylputrescine^47^, were higher in the cachexia group than in the non-cachexia group, both with and without fatigue. Polyamine metabolism is associated with cancer progression. It has been previously reported that spermine, a metabolite of the polyamine metabolic pathway, is upregulated in colorectal cancer patients with CRF^33^. In addition, salivary polyamines have been reported as potential biomarkers for the diagnosis of pancreatic and colorectal cancer^48,49^. The polyamine metabolic pathway is an important regulator of cellular proliferation and differentiation, and the disruption of polyamine homeostasis leads to oncogenic pathophysiology^50^. Polyamine metabolism is coordinately regulated by the proto-oncogene, MYC, particularly in proliferative tissues, and is further augmented in many cancer cells harboring hyperactivated MYC^51,52^. The inhibition of ornithine decarboxylase, one of the rate-limiting enzymes in the polyamine metabolic pathway, suppressed cancer aggressiveness in an in vitro study^53^. Spermidine/spermine *N*^1^-acetyltransferase 1 (SAT1), the rate-limiting enzyme in polyamine catabolism, was reported to elevate cancer aggressiveness by stimulating the expression of DNA damage response pathways and cell cycle regulatory genes in an in vitro study^54^. Spermine oxidase, an enzyme responsible for converting spermine to spermidine, actively contributes to colorectal cancer tumorigenesis, serving as an independent prognostic factor for colorectal cancer in vitro^55^.

In the study of chemotherapy-induced CRF, metabolites of cysteine and methionine metabolism, including cysteine, cystathionine, choline, and 5′-methylthioadenosine, showed lower concentrations before chemotherapy in the exacerbated fatigue group than in the non-exacerbated group (Fig. 5). Cysteine and methionine metabolism have been reported to be unique to patients with cancer and fatigue^39^. Further, the plasma metabolite levels of cysteine and methionine metabolic pathways were lower in patients with CFS and Gulf War illness^56,57^. Plasma cysteine and methionine levels also decreased after mental fatigue loading in healthy volunteers^38^. Interestingly, oxidative stress levels are elevated in patients with CFS and in fatigued animal models^58^, and *N*-acetylcysteine, an *N*-acetyl derivative of the natural amino acid, cysteine, has efficient antioxidant activity^59,60^. Our results suggest that oxidative stress is associated with CRF, including chemotherapy-induced fatigue, and that metabolites associated with antioxidation are expected to alleviate CRF.

Our results also showed that plasma citrulline levels before chemotherapy tended to be lower in the exacerbated group than in the non-exacerbated group (*P* = 0.056), and the plasma ornithine/citrulline ratio tended to be higher in the exacerbated group (*P* = 0.056). Plasma citrulline levels were reported to be lower in fatigued rats^28^. Furthermore, a previous study on patients with CFS reported that the ornithine/citrulline ratio was significantly higher than that in healthy controls^29^. If a higher ratio is involved in low detoxification capability in the liver, exacerbated CRF during chemotherapy might be induced by low detoxification.

In this study, all patients with cachexia (100%), four with non-cachexia and fatigue (80%), and two with non-cachexia without fatigue (40%) died during the observation period. The median times to death were 18, 271, and 477 days, respectively. Patients who died in the non-cachexia group had higher plasma putrescine levels than in those who had survived (*P* = 0.029). However, the levels of other plasma metabolites of the polyamine metabolic pathway did not differ significantly. Patients who died in the non-cachexia group had lower plasma cysteine levels than in those who had survived. These findings suggest that plasma levels of putrescine and cysteine are potential prognostic indicators, even in the pre-cachexia stage. Therapeutic intervention in the cysteine and methionine metabolic pathways may prolong patient survival times.

This study has several limitations. First, the sample size in this study was small and only conducted for urological cancer. Although some metabolomic analyses yielded consistent results even under FDR correction, no metabolites showed significant differences under FDR correction in the comparison between the fatigue-exacerbated and non-exacerbated chemotherapy groups, likely because of the small sample size. Based on the results of this study, large-scale, multicenter trials should be performed. Second, the subjects in this study included 14 males and one female, and we could, therefore, not investigate differences in metabolic profiles associated with sex differences. Third, we standardized the time of plasma collection before breakfast but did not analyze usual eating habits. However, given these limitations, the present study provides an essential information resource for CRF.

In conclusion, this study revealed the different metabolic profiles in patients with CRF due to other causes, including cachexia and chemotherapy. Therefore, these metabolites may serve as cause-oriented biomarkers of CRF. Such metabolic profiles provide the possibility of treating and/or preventing CRF in the process of cancer progression and improving prognosis through the intake of metabolites.

## Methods

### Ethics statement

This study was approved by the Ethics Committees of Shiga University of Medical Science (permission number: R2017‐110) and RIKEN (permission number: K2019-014) and conducted following the Declaration of Helsinki. Informed consent was obtained in written from all the participants included in the studies. All research was performed in accordance with the relevant guidelines and regulations.

### Subjects

The subjects in this study were 15 patients with advanced urological cancer who underwent anticancer treatment at the Shiga University of Medical Science Hospital from 2017 to 2020. All data in this study were obtained from five patients with and 10 without cachexia. Patients classified as having cachexia were defined as in a previous report^61^: patients showing weight loss of more than 5% a month, those showing a body mass index less than 20 kg/m^2^ and weight loss of more than 2% a month, or those showing sarcopenia and weight loss of more than 2% a month, as well as a life expectancy of fewer than three months (Supplementary Fig. S3). All non-cachectic patients received chemotherapy during the study.

Inclusion criteria required patients with urological cancer, all of whom were either diagnosed with cachexia or undergoing chemotherapy due to advanced cancer at Shiga University of Medical Science Hospital. Exclusion criteria included patients who could not answer the questionnaire owing to mental illness or retardation and those who were under 20 years of age. Patients with congenital or acquired metabolic disorders affecting the metabolome analysis data were also excluded.

### Fatigue severity assessment

We assessed fatigue severity in all subjects using the FACIT-F measure. The FACIT-F is a 13-item measure that evaluates self-reported fatigue and its impact on daily activities and functions (http://www.facit.org). FACIT-F has been reported to be reliable and valid for assessing fatigue severity in patients with diseases, including cancers^62^. In this study, the FACIT-F data were obtained from all subjects in the morning (before breakfast). Following previous reports^42,62^, patients with a total score < 43 were classified as those with fatigue and that ≥ 43 as those without fatigue. All non-cachexic patients underwent a series of chemotherapy with gemcitabine and cisplatin, carboplatin, or paclitaxel and received the scheduled regimens. FACIT-F data were obtained on the first and on seventh days of a period of chemotherapy. The median value of decreased FACIT-F scores by chemotherapy was employed for dividing the patients into the exacerbated (≥ the median value: 7 ± 12.4) and non-exacerbated groups (< the median value) of fatigue.

### Metabolomic analysis

Plasma samples were collected from all patients before breakfast on the same day after answering the questionnaires and immediately stored in a freezer at − 80 °C until measurement. For metabolome analysis, positively and negatively charged metabolites extracted from plasma samples were quantified according to a previously reported method^63^. For the positive and negative ion metabolite, 10 μL plasma sample was mixed with 90 μL methanol containing 1 μM camphor-10-sulfonic acid and 1.5 μM of each standard compound (d_8_-spermine, d_8_-spermidine, d_6_-*N*^*1*^-acetylspermidine, d3-*N*^1^-acetylspermine, d6-*N*^1^,*N*^8^-diacetylspermidine, d6-*N*^1^*,N*^12^-diacetylspermine, hypoxanthine-^13^C_2_,^15^N, and 1,6-diaminohexane). Following centrifugation at 20,380×*g* for 10 min at 4 °C, 90 μL supernatant was transferred to a fresh tube and vacuum-dried. The sample was mixed with 10 μL of 90% methanol and 190 μL water containing 20 μM of each standard (sulfanilic acid and methionine sulfone) and thereafter vortexed and centrifuged at 20,380×*g* for 10 min at 4 °C. Finally, 1 μL of the samples was used for an LC-TOF-MS.

The conditions used for the 1290 Infinity LC system and G6230B TOF-MS measurement equipment (Agilent Technologies, Santa Clara, CA, USA) and how raw data were processed using Agilent MassHunter Qualitative Analysis software (version B.08.00; Agilent) were as previously reported^64^. We also analyzed 151 standard compounds containing metabolites to determine linearity between the peak areas of the metabolites. Raw data were analyzed using typical LC-TOF-MS data processing^65^. The corresponding metabolite-derived peaks were detected in each sample. The peak areas were integrated and divided by those of the internal standards to eliminate fluctuations in MS sensitivity. By evaluating the quantification quality, we confirmed that most of the peak areas were in the linearity range and treated peaks smaller than the lower linearity limit as non-detected peaks. The absolute concentration of each metabolite was calculated based on the ratio of these values in plasma and standard mixtures.

### Data and statistical analyses

Data analyses and visualization were conducted using MetaboAnalyst (ver. 5; https://www.metaboanalyst.ca/). The heatmap visualized the Z-score values of the metabolite concentrations. Data were aligned with clustering using the Ward method and elucidation distance. PCA showed the first and second principal components (PC1 and PC2) with contribution ratios as score plots. Each plot indicates one sample and the 95% confidence interval is indicated by colored circles. Volcano plots were used to visualize the differences between the two groups. The X- and Y-axes show the log_2_ fold change of averaged concentrations and − log_10_ of the *P*-value (Mann–Whitney *U* test). Each plot represented a single metabolite. Metabolites above the horizontal line (Y > 1.3) indicate *P* < 0.05. PLS-DA was conducted to evaluate the discrimination ability of the overall metabolomic data between the two groups. The VIP scores were calculated for each metabolite. Metabolites with higher VIP values contributed significantly to the discrimination of the given groups. Pathway-level differences between the two groups were evaluated via enrichment analysis using the small-molecule pathway database (https://www.smpdb.ca/). The metabolite concentration was converted to a Z-score for PLS-DA and enrichment analyses.

The Mann–Whitney *U* test was used for unpaired, two-group comparisons. A *P* < 0.05 was considered significant for all tests. Considering multiple independent tests, the FDR using the Benjamini–Hochberg method was used for *P*-value correction. The horizontal bars in the box plots indicate 25%, 50%, and 75% of the data. The whiskers indicate 5% and 95%, respectively, and the external data are plotted. These analyses were conducted using GraphPad Prism software (v.9.5.1; GraphPad Software, San Diego, CA, USA).

Cohen’s d was a statistical measure used to quantify the size of the difference between two groups. A Cohen’s d value exceeding 0.8 indicated a substantial effect size. A Cohen’s d value was calculated by taking the difference between the means of the two groups and dividing it by a pooled standard deviation.

### Supplementary Information

Supplementary Legends.

Supplementary Figure S1.

Supplementary Figure S2.

Supplementary Figure S3.

## Data Availability

The data sets generated during the current study are available from the corresponding author on reasonable request.
