# Supplementary material for: Metabolomic profiling of cancer-related fatigue involved in cachexia and chemotherapy
Source: Sci Rep. 2024 Apr 9;14:8329. doi: 10.1038/s41598-024-57747-y (PMC11004174; doi:10.1038/s41598-024-57747-y)
Supplement: Supplementary file 1 — Supplementary Legends. [file 41598_2024_57747_MOESM1_ESM.docx]

**Supplementary data**

**Supplementary Figure S1. Plasma metabolites in the fatigued and non-fatigued groups**

Volcano plots showing differences in individual metabolite concentrations between the fatigued and non-fatigued groups. The X- and Y-axes indicate the log_2_ fold change (fatigued/non-fatigued) and –log_10_ *P*-values (Mann-Whitney U test), respectively. The *P*-values were calculated using the Mann-Whitney U test and adjusted for false discovery rate (FDR) correction. FDR-adjusted *P*-value = 0.05. F., fatigued group; N.F.: non-fatigued group.

**Supplementary Figure S2. Plasma metabolites in the fatigued and non-fatigued with non-cachexia groups**

**(a)** Volcano plots showing differences in metabolite concentrations between the fatigue without cachexia and non-fatigued groups. The X- and Y-axes indicate the log_2_ fold change (fatigued/non-fatigued) and –log_10_ *P*-values (Mann-Whitney U test), respectively. (b) Score plots of partial least squares-discriminant analysis (PLS-DA) (left-hand figure). The X- and Y-axes indicate the first and second components. Quantile normalization was performed on each sample, followed by autoscaling of the metabolite concentrations to eliminate sample-dependent bias. Red represents the fatigue group and green the non-fatigue group. Variable importance in projection (VIP) scores showing the top 15 metabolites (right-hand figure). Higher concentrations compared with that of the average are represented in red and lower concentrations in blue. (c) Metabolic pathway-based analysis showing the top 25 enriched metabolite sets. The color intensity represents *P*-values, whereas the size of the circles represents the enrichment ratio. (d) Box plots of each metabolite concentration in the tryptophan metabolic pathway. Horizontal lines of the box indicate 0, 25, 50, 75, and 100% of the data. The Y-axis indicates metabolite concentrations (μM). F., fatigue without cachexia group; N.F., non-fatigued group

**Supplementary Figure S3. Plasma metabolites in the cachexia and non-cachexia groups**

Volcano plots showing differences in individual metabolite concentrations between the cachexia and non-cachexia groups. The X- and Y-axes indicate the log_2_ fold change (cachexia/non-cachexia) and –log_10_ *P*-values (Mann-Whitney U test), respectively. The *P*-values were calculated using the Mann-Whitney U test and adjusted for false discovery rate (FDR) correction. FDR-adjusted *P*-value = 0.05. C., cachexia group; N.C., non-cachexia group.
