# Supplementary figures and images for: Metabolomic profiling of cancer-related fatigue involved in cachexia and chemotherapy
Source: Sci Rep. 2024 Apr 9;14:8329. doi: 10.1038/s41598-024-57747-y (PMC11004174; doi:10.1038/s41598-024-57747-y)

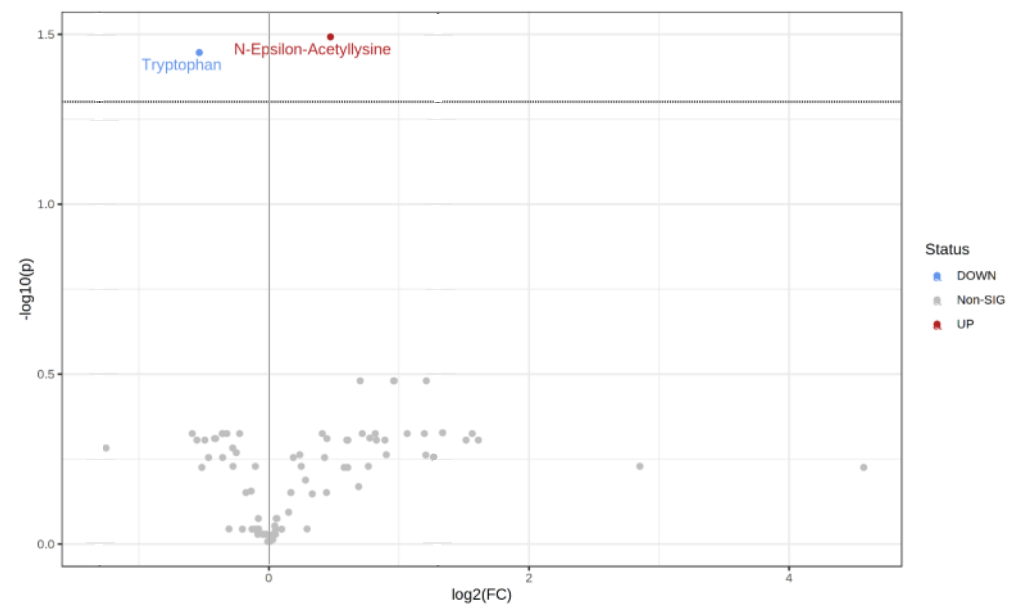

$$FC = F/NF$$

Supplement: Supplementary file 2 — Supplementary Figure S1. [file 41598_2024_57747_MOESM2_ESM.pdf]

a

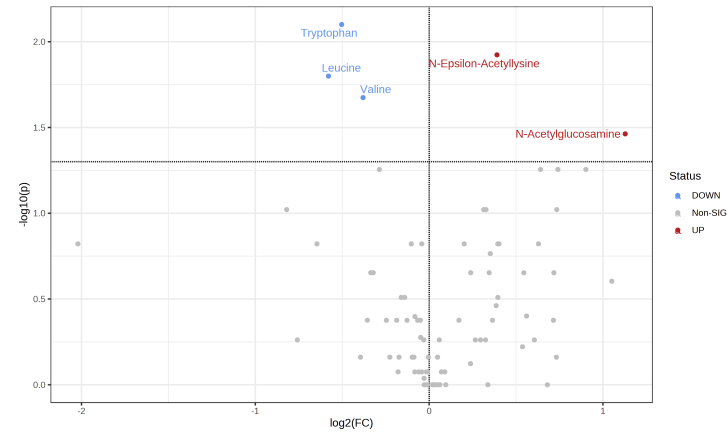

FC = F. in non-cachexia group/N.F

b

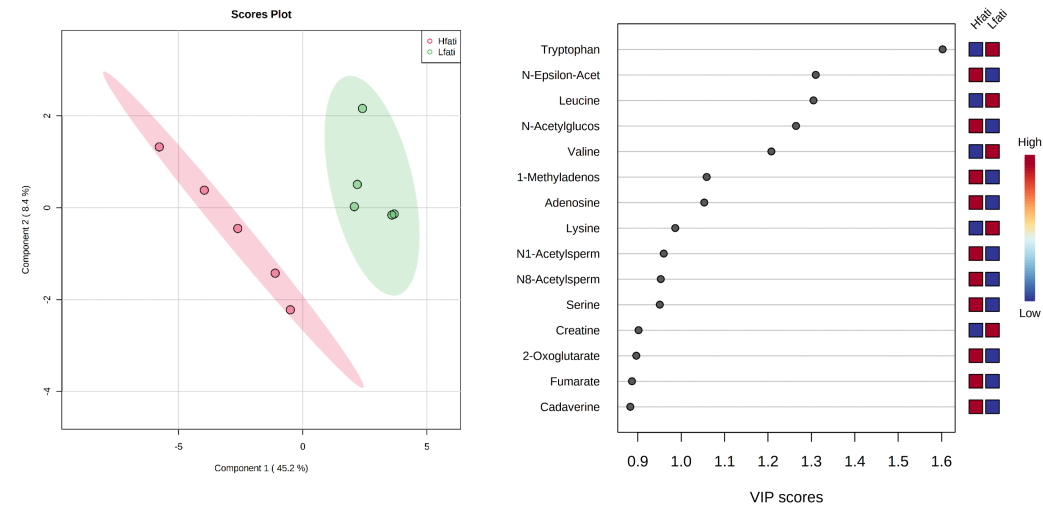

c

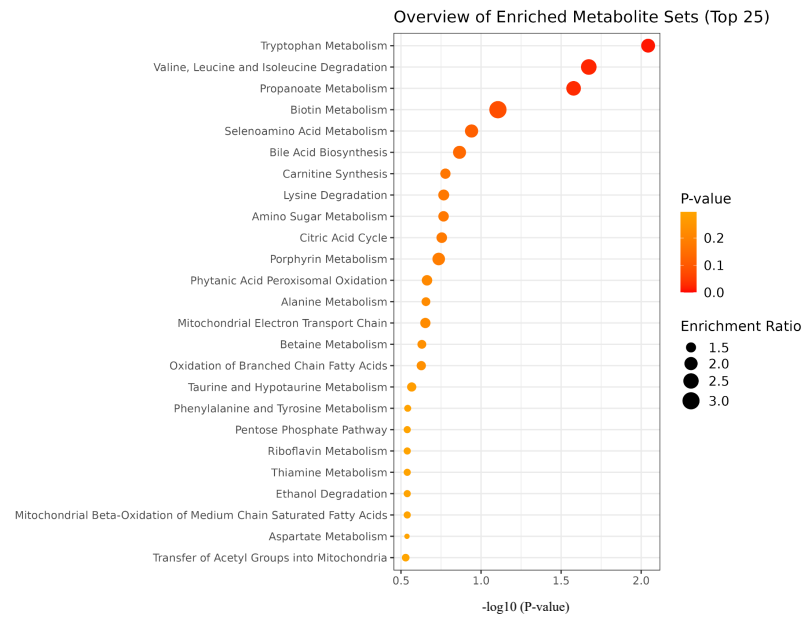

d

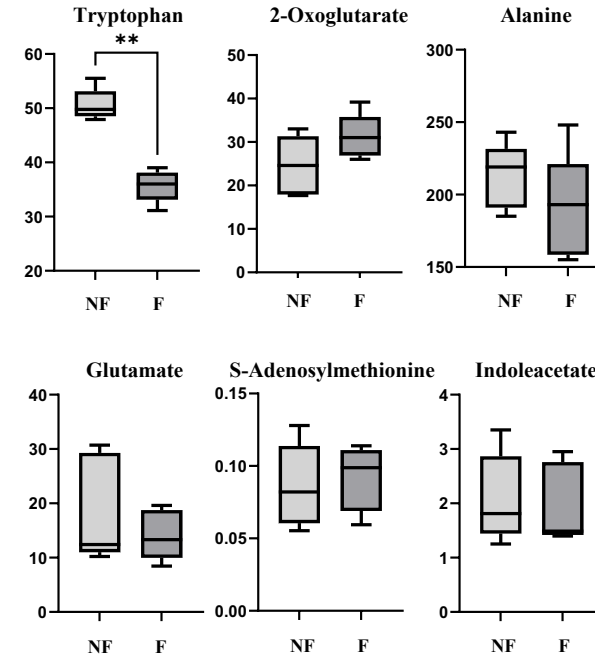

Supplement: Supplementary file 3 — Supplementary Figure S2. [file 41598_2024_57747_MOESM3_ESM.pdf]

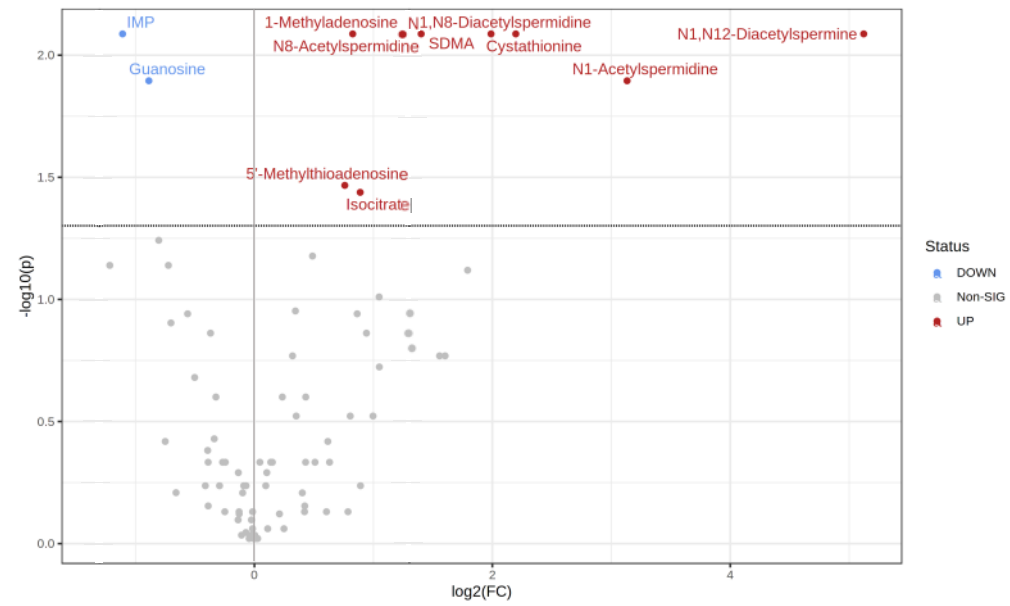

$$\text{FC} = \text{C/NC}$$

Supplement: Supplementary file 4 — Supplementary Figure S3. [file 41598_2024_57747_MOESM4_ESM.pdf]
